# Relationship between Ischemic Stroke and Pulse Rate Variability as a Surrogate of Heart Rate Variability

**DOI:** 10.3390/brainsci9070162

**Published:** 2019-07-10

**Authors:** Ajay K. Verma, Parshuram N. Aarotale, Parastoo Dehkordi, Jau-Shin Lou, Kouhyar Tavakolian

**Affiliations:** 1 School of Electrical Engineering and Computer Science, University of North Dakota, Grand Forks, ND 58202, USA; 2 Department of Electrical and Computer Engineering, University of British Columbia, Vancouver, BC V6T 1Z4, Canada; 3 Sanford Brain and Spine Center, Sanford Health, Fargo, ND 58103, USA

**Keywords:** autonomic nervous system, baroreceptor reflex, sit-to-stand test

## Abstract

Autonomic reflex ascertains cardiovascular homeostasis during standing. Impaired autonomic reflex could lead to dizziness and falls while standing; this is prevalent in stroke survivors. Pulse rate variability (PRV) has been utilized in the literature in lieu of heart rate variability (HRV) for ambulatory and portable monitoring of autonomic reflex predominantly in young, healthy individuals. Here, we compared the PRV with gold standard HRV for monitoring autonomic reflex in ischemic stroke survivors. Continuous blood pressure and electrocardiography were acquired from ischemic stroke survivors (64 ± 1 years) and age-matched controls (65 ± 2 years) during a 10-minute sit-to-stand test. Beat-by-beat heart period (represented by RR and peak-to-peak (PP) intervals), systolic blood pressure (SBP), diastolic blood pressure (DBP), and pulse arrival time (PAT), an indicator of arterial stiffness, were derived. Time and frequency domain HRV (from RR intervals) and PRV (from PP intervals) metrics were extracted. PAT was lower (248 ± 7 ms vs. 270 ± 8 ms, *p* < 0.05) suggesting higher arterial stiffness in stroke survivors compared to controls during standing. Further, compared to controls, the agreement between HRV and PRV was impaired in stroke survivors while standing. The study outcomes suggest that caution should be exercised when considering PRV as a surrogate of HRV for monitoring autonomic cardiovascular control while standing in stroke survivors.

## 1. Introduction

Impairment in autonomic function is commonly observed following ischemic stroke [1,2,3]. Autonomic cardiovascular control is a vital mechanism which regulates blood pressure during standing [4,5,6], the failure of which could cause dizziness and unexpected falls [7]. Falls that are associated with the failure to regulate blood pressure during a standing position (orthostatic intolerance), due to autonomic dysfunction, are prevalent in stroke survivors, which adversely affect their quality of life [8,9,10,11]. The prevalence of stroke increases with age, and with the anticipated increase in older persons in the future more incidents of stroke can be expected [12,13,14]. Therefore, the development of a portable and cost-effective technology for the continuous monitoring of the autonomic reflex in an ambulatory fashion can play a consequential role in the management of untoward effects of a stroke to improve the quality of life of affected persons.

Change in posture from sitting/supine to standing challenges blood pressure homeostasis due to a gravity-induced downward displacement of central blood volume [4,5]. Consequently, beat-to-beat reduction in blood pressure is sensed by the baroreceptors localized in the aortic arch and carotid sinus, the reflex i.e. a neural mediated increase in sympathetic and decrease in the vagal activity of the autonomic nervous system (ANS) causes an elevation in the heart rate and a systemic vascular resistance to ascertain blood pressure homeostasis [15,16]. Electrocardiography (ECG) derived heart rate variability (HRV), which reflects an ANS function, is widely utilized for the assessment of autonomic control of blood pressure in a non-invasive fashion [17,18,19,20].

**Figure 1:**
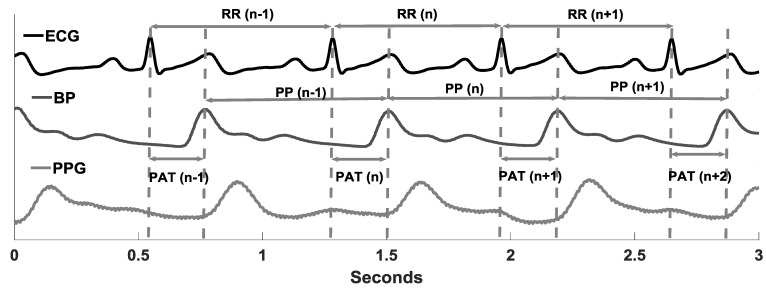
An example of simultaneously recorded ECG, finger blood pressure (BP), and finger photoplethysmography (PPG). The RR, peak-to-peak (PP), and pulse arrival time (PAT) time series were derived as shown in the figure. Finger PPG, although not utilized in this research, is shown to highlight morphological similarity with finger blood pressure waveform.

Photoplethysmography (PPG), a measure of changes in blood volume in the underlying vascular bed of tissue, provides a cost-effective and portable application for the derivation of beat-by-beat cardiac rhythm by placing a single sensor on the body and has the potential to facilitate ambulatory application [21,22,23]. A similar application by using ECG is limited due to the requirement of several electrode placements [24]. The continuously acquired blood pressure waveform is morphologically similar to PPG (Figure 1), due to the similarity in the acquisition technique, and is also utilized for the derivation of pulse rate variability (PRV) [25,26,27,28,29]. Therefore, the development of a system based on PRV for continuous, portable, and ambulatory autonomic performance monitoring can be essential for tracking the effectiveness of therapy to improve autonomic function during rehabilitation. Additionally, it can be used at home for monitoring autonomic function post-rehabilitation and could alert the user of potential falls associated with poor autonomic control of blood pressure i.e., orthostatic intolerance.

PRV is demonstrated to be a good surrogate of HRV for assessment of the autonomic function in the literature, where strong agreement between HRV and PRV is shown; primarily in young, healthy participants during a resting condition [24,27,30,31,32,33,34], while other studies have shown a decrease in the agreement under physiological conditions that perturbs cardiovascular homeostasis (tilt test, exercise) and disease [22,27,32,35,36,37,38]. This underscores the existence of inconsistencies in PRV as a surrogate of HRV with health and experimental conditions. Accordingly, a comprehensive investigation, utilizing data from a group other than young and healthy and under physiological conditions beyond resting, is warranted to gain acceptance of PRV as a surrogate of HRV for global application. In this study, we compared the degree of agreement between HRV and PRV in stroke survivors and age-matched healthy controls. Time and frequency domains metrics from the two variables (HRV or PRV) were derived and compared during a rest and orthostatic challenge applied via the sit-to-stand test. Moreover, we utilize pulse arrival time (PAT), which is inversely related to arterial stiffness [39], for the assessment of the vascular tone of the two groups. Based on the observations in the literature, we hypothesized the observation of an impairment in the agreement between HRV and PRV in stroke survivors, especially during the stand phase of the sit-to-stand test.

## 2. Materials and Methods

### 2.1. Experimental Protocol and Data Acquisition

The data utilized in this research is a part of cerebral vasoregulation in the elderly with a stroke dataset, which is publicly available on the Physionet website [40,41]. The detailed experimental protocol is summarized elsewhere [41]. Here, we briefly summarize the experimental protocol with respect to this study. Data were recorded from 41 older persons (age: 64.4 ± 1.3 years, height: 1.67 ± 0.01 m, weight: 77.6 ± 2.2 kgs, 21 females) with a history of ischemic stroke and 29 age-matched controls (age: 65 ± 2 years, height: 1.66 ± 0.01 m, weight: 76 ± 2.5 kgs, 15 females). The stroke group consisted of participants with chronic large vessel ischemic infarctions in the middle cerebral artery (MCA), which were confirmed by CT and MRI tests. At the time of recording, participants were at 6.1 ± 4.9 years (mean ± SD) from the MCA infarction and were in a clinically stable condition; their neurological and functional status were verified via their score on neurological exams i.e., the Modified Rankin Scales (MRS < 4) and the National Institutes of Health Stroke Score (NIHSS < 5).

The control group had no clinical history of stroke or other cardiovascular and/or neurological diseases. Participants with intracranial or subarachnoid hemorrhage, any acute or unstable medical conditions, and the inability to follow the details of the experimental protocol were screened out from the study. Participants with medical conditions such as diabetes, arrhythmias, and/or severe hypertension (systolic blood pressure > 200 mmHg and/or diastolic blood pressure > 100 mmHg) were also excluded from the study.

Participants enrolled in the stroke and control groups were admitted to the General Clinical Research Center (GCRC) for 2 days overnight stay. Vital signs and demographic information (height and weight) were measured by a trained GCRC nurse. Blood was drawn to obtain a lipid profile, hematocrit, and a complete blood count. Antihypertensive medications were attenuated 5-days prior to the study. The experimental protocol was approved by the research ethics board of Beth Israel Deaconess Medical Center. All participants signed an informed consent form prior to data acquisition. During the study, continuous electrocardiogram (ECG), finger blood pressure, and blood flow velocity at the MCA (Doppler ultrasound) were measured simultaneously during the sit-to-stand test. ECG was measured in a 3-lead configuration (Spacelab Medical, Issaquah, WA, USA). Blood pressure waveform was continuously recorded with Finapres (Ohmeda Monitoring Systems, Englewood, CO, USA). The data were acquired at a sampling rate of 500 Hz using Labview 6.0 and the National Instruments data acquisition system (National Instruments Inc., Austin, TX, USA).

### 2.2. Data Processing

All acquired signals were low pass filtered prior to data processing. The QRS complexes were detected from ECG using the Pan–Tompkins algorithm [42], RR interval was obtained as the time difference between two adjacent QRS complexes. Beat-by-beat systolic blood pressure (SBP) was obtained from continuous finger blood pressure as a maximum between adjacent QRS complexes. Diastolic blood pressure (DBP) was obtained from continuous finger blood pressure as a minimum value in blood pressure waveform between adjacent QRS complexes. Mean arterial pressure (MAP) was obtained as $\mathrm{MAP}\;=\;\frac{2}{3}\;\times \;\mathrm{DBP}\;+\;\frac{1}{3}\;\times \;\mathrm{SBP}$. The RR and peak-to-peak (PP) interval were obtained as a time difference between two adjacent QRS complex and blood pressure peaks, respectively. Beat-by-beat pulse arrival time (PAT) was obtained as a time difference between the blood pressure peak and the R peak of ECG for each cardiac cycle. According to the recommendation in the literature [17,18], time and frequency domain heart rate variability (HRV) and pulse rate variability (PRV) parameters were derived from the ECG and blood pressure waveform, respectively, during 5-min of sitting and 5-min of standing. The time and frequency domain parameters derived in this research are listed in Table 1 and explained here in detail [17].

The RR and PP time series were interpolated using spline interpolation to create an evenly sampled signal and resampled to 10 Hz with zero mean prior to frequency domain analysis of HRV and PRV. The Welch power spectral density (PSD) of RR and PP was calculated in very low frequency (VLF, 0–0.04 Hz), low frequency (LF, 0.04–0.15 Hz), and high frequency (HF, 0.15–0.4 Hz) bands [17]. Then normalized power in the LF and HF bands was calculated as $\mathrm{LF}\;\left(n.u.\right)\;=\;\frac{\mathrm{LF}}{\left(\mathrm{LF}\;+\;\mathrm{HF}\right)}$, $\mathrm{HF}\;\left(n.u.\right)\;=\;\frac{\mathrm{HF}}{\left(\mathrm{LF}\;+\;\mathrm{HF}\right)}$, and from the two indices the $\frac{\mathrm{LF}}{\mathrm{HF}}$ ratio was calculated. The PSD was computed with a Hamming window of size with 256 samples and 50% overlap. The Pearson correlation coefficient (R) was computed to underscore the degree of agreement between HRV and PRV for the respective parameters for the two groups (control/stroke) under two conditions (sit/stand). Data analysis was performed using MATLAB (MathWorks Inc., Natick, MA, USA).

### 2.3. Statistical Analysis

Two-factor test of analysis of variance (ANOVA) was performed to highlight the differences in the cardiovascular parameters (RR intervals, blood pressure, and PAT) with the condition (sit/stand) and group (control/stroke). One-Factor ANOVA was conducted to account for the difference which may exist between HRV and PRV. Results were considered significant at α = 0.05. However, given the limited sample size of the study, the results at α = 0.10 are also discussed to highlight possible trends. The statistical analysis was performed using IBM SPSS Statistics 23 software (IBM Corporation, Armonk, NY). The results are presented as mean ± SE unless mentioned otherwise.

## 3. Results

To perform pairwise HRV and PRV comparisons, 29 controls and 29 stroke survivors (12 left side, 12 right side, 4 bilateral MCA infarction, and 1 unknown) were analyzed (first 29 of the group). The signal morphology and the calculation of beat-by-beat RR, PP, and PAT using ECG and blood pressure waveforms are outlined in Figure 1.

**Figure 2:**
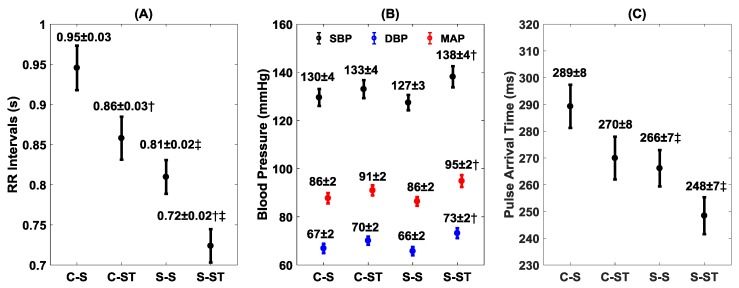
The behavior of RR intervals (**A**), blood pressure (**B**), and PAT (**C**) with group (control/stroke) and condition (sit/stand). The figure presents the distribution (mean ± SE) of control–sit (C–S), control–stand (C–ST), stroke–sit (S–S), and stroke–stand (S–ST). † represents a significant difference from sit (same group), while ‡ represents a significant difference from control (same condition).

The mean values of RR intervals and blood pressure for the two groups under the two conditions are summarized in Figure 2A,B, respectively. The RR intervals decreased significantly in both groups during standing compared to sitting (Figure 2A). The stroke group had a lower value for RR intervals during sitting (*p* < 0.001) and standing (*p* < 0.001) compared to controls. The stand test had no effect on blood pressure (SBP, *p* = 0.51; DBP, *p* = 0.22; MAP, *p* = 0.29) in controls (Figure 2B). However, the blood pressure increased (SBP, *p* = 0.04; DBP, *p* = 0.006; MAP, *p* = 0.007) in the stroke group during standing compared to sitting. The pulse arrival time (PAT) was lower both during sitting (*p* = 0.03) and standing (*p* = 0.04) in the stroke group compared to controls (Figure 2C); the stand test caused a decrease in PAT in both controls (*p* = 0.07) and stroke survivors (*p* = 0.09) (Figure 2C).

**Figure 3:**
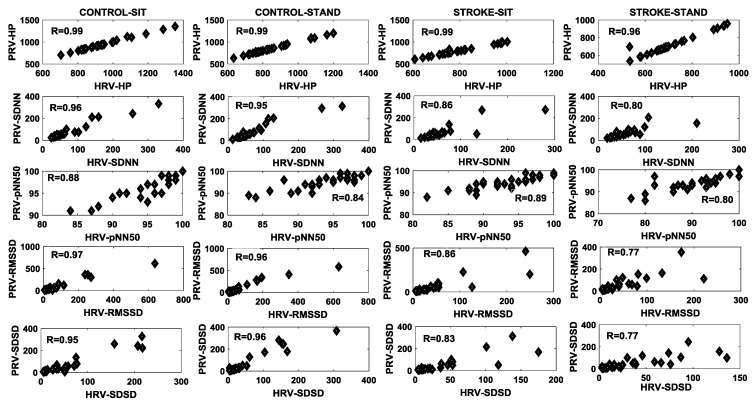
The degree of agreement, determined by computing the Pearson correlation coefficient, between HRV and PRV for different time domain features for the two groups (control/stroke) and conditions (sit/stand).

The correlation between HRV and PRV for different time domain features is summarized in Figure 3. A high correlation (R > 0.95) for the heart period was observed in both groups under both conditions. A deterioration in the correlation between HRV and PRV was observed in the stroke group compared to controls during sitting, and more so during standing for SDNN, RMSSD, and SDSD (Figure 3). Only a minor fluctuation in the correlation between the group and condition was observed for pNN50 (Figure 3).

**Figure 4:**
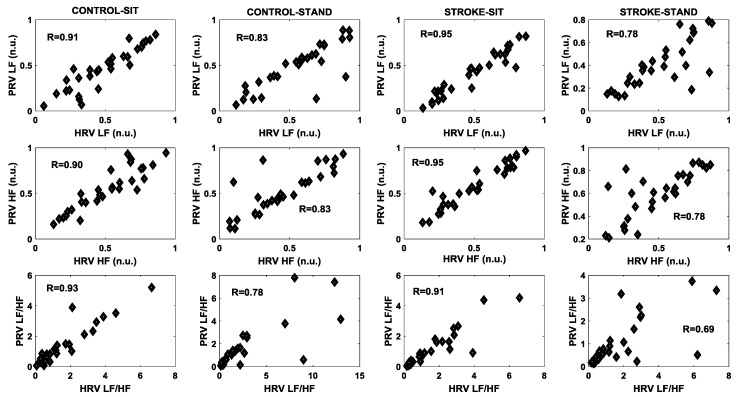
The degree of agreement, determined by computing the Pearson correlation coefficient, between HRV and PRV for different frequency domain features for the two groups (control/stroke) and conditions (sit/stand).

In the frequency domain, the correlation was relatively higher during sitting compared to standing both in controls as well as in stroke survivors for LF, HF, and LF/HF (Figure 4). The stand test had a more adverse effect on stroke group compared to controls for LF (R = 0.78 vs. R = 0.83), HF (R = 0.78 vs. R = 0.83), and LF/HF (R = 0.69 vs. R = 0.78).

**Figure 5:**
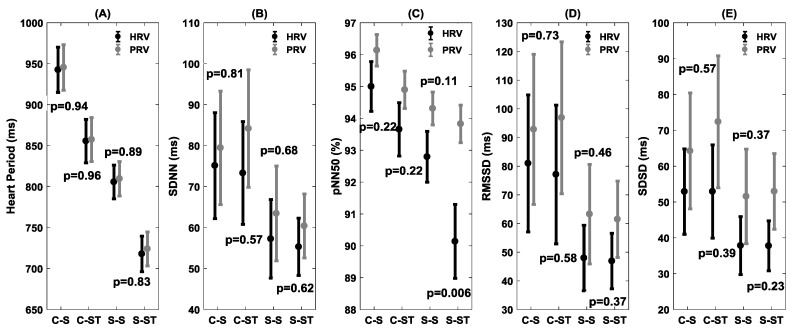
Statistical comparison of HRV and PRV for two groups (control/stroke) and two conditions (sit/stand) for different time domain measures i.e., heart period (**A**), SDNN (**B**), pNN50 (**C**), RMSSD (**D**), and SDSD (**E**). The figure represents the distribution (mean ± SE) in control–sit (C–S), control–stand (C–ST), stroke–sit (S–S), and stroke–stand (S–ST). The figure also lists the statistical p-value of the comparison.

**Figure 6:**
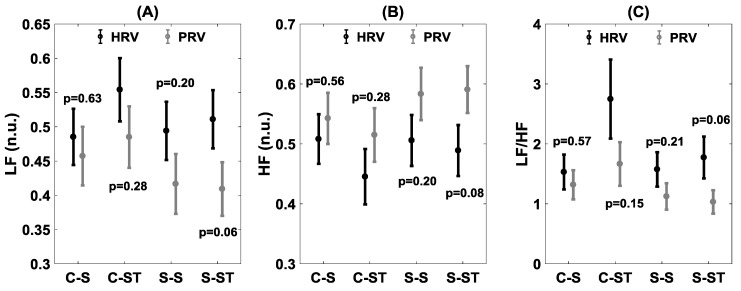
Statistical comparison of HRV and PRV for the two groups (control/stroke) and the two conditions (sit/stand) for normalized LF (**A**), normalized HF (**B**), LF/HF ratio (**C**). The figure represents the distribution (mean ± SE) in control–sit (C–S), control–stand (C–ST), stroke–sit (S–S), and stroke–stand (S–ST). The figure also lists the statistical *p*-value of the comparison.

The statistical comparison between HRV and PRV for both time and frequency domains is summarized in Figure 5 and Figure 6, respectively. In the time domain, the statistical difference between HRV and PRV for pNN50 (*p* = 0.006) was observed in the stroke group during the standing phase (Figure 5C). In the frequency domain, the differences between HRV and PRV were observed during the standing phase in the stroke group for LF (*p* = 0.06), HF (*p* = 0.08), and LF/HF (*p* = 0.06) (Figure 5).

## 4. Discussion

Autonomic dysfunction is an adverse symptom experienced by stroke survivors and is majorly accountable for orthostatic intolerance and associated falls leading to incapacitation and poor quality of life. Pulse rate variability has the potential for a cost-effective, portable, and ambulatory application for continuous monitoring of autonomic cardiovascular control in stroke survivors, which can assist in the management and mitigation of debilitating effects of stroke. PRV is shown to be a good surrogate of HRV for monitoring cardiovascular control in young, healthy participants during a stationary condition (rest), however, its potential towards monitoring autonomic reflex in people with a history of neurological disorder under the physiological condition challenging cardiovascular homeostasis (such as standing) is not well investigated and requires special attention. In this article, we investigated the effect of ischemic stroke, a neurological condition known to adversely affect autonomic function, on the viability of PRV as a surrogate of HRV for monitoring autonomic cardiovascular control while standing. The primary finding of the study was a reduction in the agreement (both in time and frequency domains) between HRV and PRV during standing in the stroke survivors. The analysis outcome suggests that caution should be exercised when considering PRV as a surrogate of HRV for monitoring autonomic reflex during orthostatic challenges in the stroke survivors.

To assess cardiovascular control, the alterations in RR intervals, SBP, DBP, and MAP were noted between the two groups during the sit-to-stand test. While both control (*p* = 0.012) and stroke (*p* = 0.014) groups showed decrease in RR intervals (increase in heart rate) during the standing phase when compared to sitting, the stroke group had lower values for RR intervals (higher heart rate) while sitting (*p* < 0.001) as well as standing (*p* < 0.001) compared to controls. This suggests the resetting of arterial baroreceptors as a ramification of ischemic infarction, resulting in a higher resting heart rate. Reduced physical activity in stroke survivors could also have contributed towards a resetting of the resting heart rate to a higher value. No difference (*p* > 0.10) was observed in the sitting or standing blood pressure values between the control and stroke groups (Figure 2); nevertheless, with the application of stand test elevation in blood pressure (SBP, *p* = 0.04; DBP, *p* = 0.006; MAP, *p* = 0.007) values were observed only in the stroke group (Figure 2). This hints toward the different vascular reflexes which regulate cardiovascular function in stroke survivors compared to controls during the standing phase. Analysis of autonomic blood pressure regulatory controls via arterial baroreflex sensitivity can shed further light in this regard.

The potential of PRV to be a surrogate of HRV is dependent on the peripheral blood flow, where the elasticity and stiffness of arteries plays a consequential role. In this regard, we utilized the concept of pulse arrival time (PAT), a marker of arterial stiffness, to highlight vascular alterations between the groups under two conditions [39,43]. The stroke group had lower (266 ± 7 ms vs. 289 ± 7 ms, *p* = 0.03) PAT values at rest compared to controls; this observation further highlights altered vascular behavior compared to controls 6.1 ± 4.9 years (mean ± SD) post-incident. Further, during the stand test, while both control (270 ± 7 ms vs. 289 ± 7 ms, *p* = 0.07) and stroke (248 ± 7 ms vs. 266 ± 7 ms, *p* = 0.09) groups showed a decrease in PAT as a result of an increase in heart rate, the PAT in the stroke group was lower (248 ± 7 ms vs. 270 ± 7 ms, *p* = 0.04) compared to controls while standing (Figure 2C), which further underscored the differences in vascular behavior between the groups. Arterial stiffness is a recognized risk factor for stroke [44]; lower PAT values in stroke survivors emphasize the existence of residual effects of stroke on the vascular tone and could affect the blood flow, which may cause disagreement between the electrical (ECG) and mechanical (BP waveform) systems for obtaining beat-by-beat cardiac rhythm and requires future comprehensive investigation.

In accordance with the literature, we observed high agreement between the ECG and the blood pressure derived heart period for both controls and stroke groups during the sitting phase (Figure 3). During the standing phase, the correlation between the ECG and the blood pressure derived heart period remain unchanged (>0.99) for controls; however, the correlation was reduced in the stroke group (Figure 3). Nevertheless, no statistical difference was observed in the ECG or the blood pressure derived heart period for either group during the two conditions (Figure 5A). This suggests that the effect of healthy aging or neurological disorder is not likely to influence the capability of acquiring heart period (or heart rate) from blood pressure waveforms during orthostatic challenge.

Further, the stroke group showed lower agreement between the ECG and the blood pressure derived SDNN, where lower correlation during both sitting (R = 0.86 vs. 0.96) and standing (R = 0.80 vs. 0.95) was observed in the stroke group compared to controls; this further accentuated the loss rhythmic beat-by-beat fluctuation to ascertain cardiovascular homeostasis. Similarly, RMSSD in the stroke group during both sitting (R = 0.86 vs. 0.97) and standing (R = 0.77 vs. 0.97) exhibited lower agreement compared to controls. The ECG and the blood pressure derived pNN50 was lower in both groups during both conditions, suggesting that pNN50 is likely to be affected by both aging and neurological conditions, therefore, blood pressure derived pNN50 is not a good indicator for autonomic cardiovascular control in such a group.

Frequency domain analysis of HRV and PRV can quantify the sympathetic activity (LF (n.u.)), vagal or parasympathetic activity (HF (n.u.)), as well as the sympathovagal balance (LF/HF), accordingly, providing vital information pertaining to the ANS influence on cardiovascular control under different physiological conditions [17]. A high correlation (>0.95) between HRV and PRV during the sitting phase was observed for both groups in the LF (n.u.), HF (n.u.), and LF/HF ratio. However, under the influence of a orthostatic challenge (standing), the correlation between HRV and PRV was reduced both in controls as well as in stroke groups with a more profound reduction in the stroke group, especially for the LF/HF ratio (R = 0.69 vs. R = 0.78), see Figure 4. This observation suggests PRV derived frequency domain parameters may not reliably indicate the dynamics of autonomic cardiovascular control during an orthostatic challenge and caution should be exercised while utilizing PRV for the assessment of autonomic cardiovascular control during orthostatic challenges beyond a young, healthy population.

The comparison of HRV and PRV via statistical analysis exhibited behavior analogous to correlation analysis. In the time domain, for pNN50, a significant difference (*p* = 0.006) between HRV and PRV was observed for the stroke group during the standing phase while in the control group no difference (*p* = 0.22) for pNN50 was observed during the standing phase. During the sitting phase, although the correlation between HRV and PRV was reduced in the stroke group compared to controls in the time domain, no statistical difference (*p* > 0.10) in the time domain was observed between HRV and PRV in the stroke group. In the frequency domain, no difference (*p* > 0.10) was observed between HRV and PRV during the sitting or standing phases. However, in the stroke group, although there was no difference (*p* > 0.10) between HRV and PRV during the sitting phase, a difference was observed for LF (*p* = 0.08), HF (*p* = 0.08), and the LF/HF (*p* = 0.06) ratio during the standing phase (Figure 6). This observation led us to conclude that PRV fails to accurately reflect the sympathetic, parasympathetic, or sympathovagal balance in the stroke group during standing.

## 5. Conclusions and Limitations

In conclusion, we observed disagreement between HRV and PRV in the stroke survivors during the sitting phase (time domain via correlation analysis) as well as during the standing phase (frequency domain via correlation and statistical analysis) compared to controls. This finding leads us to conclude that PRV may not be a reliable surrogate of HRV for monitoring autonomic cardiovascular control while standing, in people with a history of ischemic stroke. A tremendous amount of literature is focused on quantifying the relationship (HRV vs. PRV) in young, healthy participants during resting conditions, while when the same comparative relationship was studied in an unhealthy population, unfavorable outcomes were observed [36,37,38]. Therefore, the system developed for monitoring effectiveness of therapy to improve orthostatic tolerance in a rehabilitation center as well as for self-monitoring of autonomic function at home post-rehabilitation based on PRV may be limited in effect in mitigating falls related to orthostatic intolerance. Unfortunately, limited study exists which extends the HRV and PRV comparison beyond a young, healthy population [45]. Accordingly, a comprehensive investigation including the population with cardiovascular and/or neurological diseases is required to ensure the global application of PRV for monitoring autonomic cardiovascular control with an aim to mitigate the deleterious effect of aging or neurological disorder on day-to-day activity to improve the quality of life. Moreover, the vascular tone of the study group should also be accounted for to further our understanding regarding the agreement between electrical (electrocardiography) and mechanical (blood pressure or photoplethysmography) signals utilized for the assessment of autonomic cardiovascular control.

The limitation of the present work was the unavailability of simultaneous young, healthy data under orthostatic challenge. Although it is demonstrated in the literature that a high degree of agreement exists between the HRV and PRV during orthostatic challenges in young, healthy participants, it would be beneficial to compare the three groups under the same experimental protocol to quantify the degree of change in PRV associated with healthy aging and neurological conditions. Further, it should be noted that PRV in this study was derived from continuous blood pressure waveform and not PPG, as due to the similarity in blood pressure waveform and PPG (Figure 1), it was expected that the conclusion would be analogous.

## Figures and Tables

**Table 1:** Time and frequency domain metrics utilized in this research to compare the agreement between heart rate variability (HRV) and pulse rate variability (PRV).

HRV/PRV Variables	Domain
Mean RR/PP intervals (heart period, HP)	Time
Standard deviation of RR/PP intervals (SDNN)	Time
Root mean square of successive difference between adjacent intervals (RMSSD)	Time
Standard deviation of successive difference between adjacent intervals (SDSD)	Time
Percentage of adjacent RR/PP intervals that differ by more than 50 ms (pNN50)	Time
Normalized Low-Frequency (LF (n.u.))	Frequency
Normalized High-Frequency (HF (n.u.))	Frequency
LF/HF Ratio	Frequency
